# Modulating intestinal neuroimmune VIPergic signaling attenuates the reduction in ILC3-derived IL-22 and hepatic steatosis in MASLD

**DOI:** 10.1097/HC9.0000000000000528

**Published:** 2024-10-17

**Authors:** Henry H. Nguyen, Jhimmy Talbot, Dayi Li, Varsha Raghavan, Dan R. Littman

**Affiliations:** 1 Department of Cell Biology, New York University School of Medicine, New York, New York, USA; 2 Department of Medicine and Department of Microbiology, Immunology, and Infectious Diseases, Snyder Institute for Chronic Diseases, Cumming School of Medicine, University of Calgary, Calgary, Alberta, Canada; 3 Basic Sciences Division, Fred Hutchinson Cancer Center, Seattle, Washington, USA; 4 Howard Hughes Medical Institute, New York, New York, USA

**Keywords:** NAFLD, host immune response, neuroimmune interactions, gut-liver axis

## Abstract

**Background:**

Metabolic dysfunction–associated steatotic liver disease (MASLD, formerly known as NAFLD) is a major driver of cirrhosis and liver-related mortality. However, therapeutic options for MASLD, including prevention of liver steatosis, are limited. We previously described that vasoactive intestinal peptide–producing neurons (VIP-neurons) regulate the efficiency of intestinal dietary fat absorption and IL-22 production by type 3 innate lymphoid cells (ILC3) in the intestine. Given the described hepatoprotective role of IL-22, we hypothesize that modulation of this neuroimmune circuit could potentially be an innovative approach for the control of liver steatosis.

**Methods:**

We used a model of diet-induced MASLD by exposing mice to a high-fat diet (HFD) for 16 weeks, when the development of liver steatosis was first observed in our animals. We characterized IL-22 production by intestinal ILC3 at this dietary endpoint. We then evaluated whether communication between VIP-neurons and ILC3 affected IL-22 production and MASLD development by exposing mice with a conditional genetic deletion of *Vipr2* in ILC3 (*Rorc(t)* ^*Cre*^*Vipr2* ^*fl/fl*^) to the HFD. We also performed intermittent global inhibition of VIP-neurons using a chemogenetic inhibitory approach (*Vip* ^*Ires-Cre*^ *hM4Di* ^*LSL*^) in HFD-fed mice.

**Results:**

Production of IL-22 by intestinal ILC3 is reduced in steatotic mice that were exposed to an HFD for 16 weeks. Targeted deletion of VIP receptor 2 in ILC3 resulted in higher production of IL-22 in ILC3 and was associated with a significant reduction in liver steatosis in mice under HFD. Global inhibition of VIP-producing neurons also resulted in a significant reduction in liver steatosis.

**Conclusions:**

Modulating VIPergic neuroimmune signaling can ameliorate the development of hepatic steatosis induced by a surplus of fat ingestion in the diet. This neuroimmune pathway should be further investigated as a potential therapeutic avenue in MASLD.

## Graphical abstract

**Figure:**
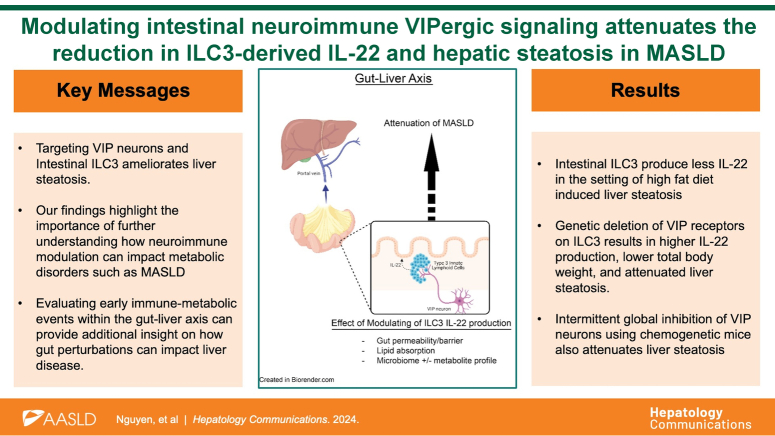

## INTRODUCTION

Metabolic dysfunction–associated steatotic liver disease (MASLD, formerly known as NAFLD) describes a spectrum of diseases ranging from liver steatosis to liver inflammation, liver fibrosis, and end-stage cirrhosis. Its prevalence is increasing globally, paralleling obesity, diabetes, and dyslipidemia.^1^,^2^,^3^ Current medical interventions focus on lifestyle modifications through diet and exercise, with adjunct pharmacologic therapeutic options for the treatment of MASLD being an active area of research.^4^ With limited therapy and many investigational drugs having recently failed in clinical trials, there is an ongoing unmet need for effective medical therapy to complement lifestyle changes aimed at reducing liver steatosis and overall disease progression.

Recent studies suggest a crucial role of the intestinal immune system in the pathogenesis of MASLD, highlighting the importance of the gut-liver axis and immune signals for host metabolic homeostasis.^5^,^6^,^7^ Among these immune-metabolic signals, the cytokine IL-22 has been illustrated to play a vital role in metabolic conditions, including that of MASLD.^8^,^9^,^10^,^11^ IL-22 is a member of the IL-10 family of cytokines and plays a central role in maintaining gastrointestinal epithelial barrier integrity,^12^ which has been implicated in the pathogenesis of MASLD.^5^,^13^ Systemic treatment with IL-22 can ameliorate hepatic steatosis in preclinical models of MASLD and can reduce serum levels of triglycerides in human subjects.^11^,^14^ Moreover, it has been demonstrated that IL-22 is hepatoprotective,^15^,^16^ and reduces intestinal lipid metabolism and uptake.^17^,^18^,^19^ This suggests that strategies to promote the upregulation of intestinal IL-22 may benefit patients diagnosed with MASLD.^5^,^9^,^10^,^11^

Under homeostasis, type 3 innate lymphoid cells (ILC3) are one of the main producers of IL-22 in the intestine.^20^ We recently identified in mice a neuroimmune circuit that downregulates IL-22 production by intestinal ILC3 during feeding but not during fasting.^18^ This neuroimmune circuit is formed by the interaction between vasoactive intestinal peptide (VIP)-producing neurons and ILC3. During feeding, VIP-producing neurons are activated, and the released VIP acts on the VIP receptor 2 (VIPR2) in ILC3, inhibiting IL-22 production by these cells. We observed that neuronal-mediated reduction in IL-22 levels during feeding increases the efficiency of fat uptake from the diet. Moreover, blockage of activation of VIP-producing neurons, or conditional removal of VIPR2 specifically from ILC3, reduces the efficiency of fat absorption from the diet. Here, we hypothesized that modulation of this intestinal neuroimmune circuit could alter the development of MASLD through the regulation of IL-22 by ILC3. Using both conditional genetic inactivation of VIPR2 in ILC3 and chemogenetic-mediated inhibition of VIP-producing neurons, we found that hepatic steatosis can be attenuated by modulation of VIPergic signaling in innate lymphoid cells. This suggests that strategies to prevent VIPergic activation and intestinal neuronal modulation might ameliorate MASLD development in the clinical setting.

## METHODS

### Mice

C57BL/6 mice were purchased from Taconic Biosciences. All transgenic mice were bred and maintained in the Alexandria vivarium (New York University School of Medicine) in specific pathogen-free conditions (with microbiota that includes segmented filamentous bacteria) on a C57BL/6J background. Inhibitory VIPergic neuron DREADD (Designer Receptors Exclusively Activated by Designer Drugs) mice were generated by breeding *hM4Di* ^*LSL*^ mice (*Rosa26*-*hM4Di* ^*fl-stop-fl*^ or CAG-LSL-Gi-DREADD, Jax #026219) and *VIP* ^*IRES-Cre*^ mice (B6J.Vip-IRES-Cre, Jax #031628) from Jackson Laboratories and was described previously.^18^ *RORc(t)* ^*Cre*^ and *Vipr2* ^*fl/fl*^ mice were generated in our laboratory as described^18^,^21^ and were interbred to generate *RORc(t)* ^*Cre*^*Vipr2* ^*fl/fl*^ mice (*Vipr2* ^*ΔILC3*^). Details of the genetic engineering, including target guide RNA sequences, can be found in our prior study.^18^ All mice were 6–8 weeks old at the time of treatment/diet initiation. All animal procedures were performed in accordance with the protocols approved by the Institutional Animal Care and Usage Committee of New York University School of Medicine or the NIAID, as applicable. Measurements of body and gonadal adipose tissue weight were carried out independently by blinded observers on numerically coded mice.

### MASLD preclinical model

Both C57BL/6 and transgenic mice were fed either a standard diet (normal diet or ND) or high-fat diet (HFD) containing 60% Kcal of fat (derived from lard and soybean oil), 20% Kcal of protein (derived from casein and cystine), and 20% Kcal of carbohydrate (Research Diets D12492) up to a 16-week endpoint when liver steatosis was consistently observed on histopathology. Mice were fed ad libitum with a weekly refill of dietary content and free access to water.

### VIPergic neuronal inhibition using DREADD mice

To perform global chemogenetic inhibition of VIPergic neurons in DREADD mice (*VIP* ^*IRES-Cre*^*hM4Di* ^*LSL/+*^), the animals were initiated on either ND or HFD and after 1 week of acclimatizing to the diet received treatment with Compound 21 (C21; R&D Systems Cat#5548) at a dose of 1 mg/kg through i.p. injection every 48 hours for 16 weeks. This dose and frequency were chosen based on reported pharmacokinetic data.^22^ C21 was prepared using sterile PBS, and injection was done under sterile conditions with an alcohol cleanse of the abdomen before injection. Both C21-treated and PBS-treated *VIP* ^*IRES-Cre*^*hM4Di* ^*LSL/+*^ mice, on either HFD or ND, were cohoused to minimize the potential confounding batch effect of the microbiota.

### Antibodies

We used the following antibodies for flow cytometry: anti-mouse CD3e PerCP-Cyanine 5.5 (Clone 145-2C11, Tonbo Biosciences), anti-mouse CD19 PerCP-Cyanine 5.5 (Clone 1D3, Tonbo Biosciences), anti-mouse CD11b PerCP-Cyanine 5.5 (Clone m1/70, eBioscience), anti-mouse CD11c PerCP-Cyanine 5.5 (Clone N418, eBioscience), anti-mouse CD14 PerCP-Cynanine 5.5 (Clone Sa2-8, eBioscience), rat anti-mouse CD90.2 FITC (Clone 30-H12, BD Biosciences), anti-mouse CD127 PE (Clone A7R34, BioLegend), rat anti-mouse CD196 BV421 (Clone 140706, BD Biosciences), anti-human/mouse IL-22 APC (Clone Il22JOP, eBioscience), anti-mouse KLRG1 PerCP-eF710 (Clone 2F1, eBioscience), anti-mouse TCR beta PerCP-Cyanine 5.5 (Clone H57597, eBioscience), anti-mouse NK1.1 PerCP-Cyanine 5.5 (Clone PK136, eBioscience), and anti-mouse CD16/CD32 (Clone 2.4G2, BioXcell).

### Isolation of lamina propria lymphocytes from the small intestine

The ileum (distal 14 cm of the small intestine) was carefully dissected from mice with the removal of all attached mesenteric fat tissue and Peyer’s patches. Intestinal tissue was opened and extensively cleaned of fecal matter in cold PBS. The tissue was sequentially treated with HBSS 1× (1 mM Dithiothreitol) at 37°C for 10 minutes with gentle shaking (200 rpm) and twice with 5 mM EDTA at 37°C for 10 minutes to remove epithelial cells. The remaining tissue was then minced with scissors and dissociated in Roswell Park Memorial Institute (RPMI) medium containing 10% FBS, dispase (0.05 U/mL; Worthington), collagenase (1 mg/mL Collagenase II; Roche), and DNase I (100 μg/mL; Sigma) at 37°C for 45 minutes with shaking at 175 rpm. The digested tissue was then filtered through a 70 μm strainer to remove any remaining large debris. Viable lamina propria lymphocytes were collected at the interface of a 40%/80% Percoll/Roswell Park Memorial Institute (RPMI) medium gradient (GE Healthcare) for subsequent antibody staining.

### Flow cytometry

Single-cell suspensions from the ileal lamina propria were pelleted and resuspended with surface-staining antibodies in PBS with 2% FBS and 1 mM EDTA. In brief, cell suspension was treated with F_C_ block for 30 minutes at 4°C using anti-CD16/32 Ab. Surface staining was completed at 4°C in the dark for 30 minutes. Live/dead fixable blue (ThermoFisher) was used to exclude dead cells. Monoclonal antibodies for surface staining of ILC3 include CD90.2, CD196 (CCR6), CD127 (IL-7Rα), CD14, CD19, CD11c, Cd11b, KLRG1, TCRβ, CD3, and NK1.1 (details listed above). ILC3-derived IL-22 production was evaluated by incubating cells for 4 hours at 37°C in Roswell Park Memorial Institute (RPMI) medium with 10% FBS and GolgiPlug (BD), staining for the surface markers, and, following fixation/permeabilization for 20 minutes (BD Cytofix/Cytoperm; Cat# 554714), intracellular staining with anti-IL-22 for 45 minutes at room temperature in the dark. Flow cytometric analysis was performed on an LSR II (BD Biosciences), and data were analyzed using FlowJo software version 10 (Tree Star).

#### Gating strategy

Total lymphocytes were gated based on forward and side scatter, followed by singlet cells. ILC3s were identified as the population of live lymphocytes negative for lineage markers CD14, CD19, CD11c, CD11b, KLRG1, TCRβ, TCRγ, CD3, and NK1.1 (denoted as Lineage negative or Lin−), and positive for CD127 (IL-7Rα). Among this population of cells, Thy1.2 (CD90.2) and CCR6 (CD196)-positive cells, which are the ILC3 cells of interest, were identified. The frequency of IL-22 producers was then analyzed among CCR6^+^CD90.2^+^ cells.

### Histology and Oil Red O stains

Liver samples were fixed in 4% paraformaldehyde (Electron Microscopy Science) for 24 hours or flash-frozen in an Optimal Cutting Temperature compound (Sakura) using a 2-methylbutane (Sigma 27758) bath with liquid nitrogen. Paraformaldehyde-fixed tissues were processed with paraffin embedding followed by 5 µM sections and staining using hematoxylin and eosin as per standard protocol by the Experimental Pathology Core Laboratory at New York University. Oil Red O staining was carried out with frozen sections from OCT blocks using standard protocol by the Experimental Pathology Core Laboratory at New York University. Evaluation of liver steatosis and quantification of Oil Red O deposition was carried out independently by 2 blinded researchers using coded slides. The imaging data were processed and analyzed using the ImageJ software (NIH). Oil Red O quantification was determined by evaluating 3 random areas of each liver specimen to ensure adequate coverage and representative scoring. The total red-stained areas were expressed as a percentage of the total tissue area for each region scored and compared across all groups.

### Serology

Blood collection was done under general anesthesia (ketamine 100 mg/kg and xylazine 15 mg/kg) through the portal vein after laparotomy was performed. The blood was centrifuged with the serum collected and flash-frozen in liquid nitrogen. The serum samples were sent to IDEXX BioAnalytics Laboratories, where ALT, AST, ALP, GGT, bilirubin, and random glucose were measured as per company protocol.

### Glucose tolerance test

This test was carried out as detailed.^23^ In brief, mice were fasted for 16 hours. Sterile 30% glucose (MilliporeSigma; Cat#D9434) solution was injected i.p. (1 g/kg). The i.p. route was chosen to minimize stress to the animals, ensure consistency of glucose delivery, and avoid the need for anesthesia, all of which could impact the animals’ blood glucose levels.^24^ All blood was collected through transverse nicking of the tail vein. After glucose administration, the mice were bled in sequence, and blood glucose was measured (Bayer; Contour Next Glucometer) at intervals 0 (fasting), 15, 30, 45, 60, and 120 minutes. The AUC of serum glucose as a function of time was calculated and used as a measure of systemic glucose tolerance when comparing experimental groups.

### 16S microbiota analysis in cecum fecal samples

Cecal contents were collected at the endpoint of the experiment after laparotomy was performed. The cecum was chosen to ensure a consistent site and access to stool content across all experimental groups. 16S rRNA evaluation was performed as described^25^ using a MiSeq instrument (Illumina). The 16S rRNA sequence data were processed and analyzed using QIIME v2.^26^ The 16S data has been uploaded onto bioproject PRJNA1157979.

### Statistical analysis

Both paired and unpaired nonparametric statistical analysis was used where appropriate in this manuscript. Statistical evaluation included the Mann-Whitney test, Kruskal-Wallis/one-way ANOVA on ranks, and Wilcoxon tests. This was carried out on Prism Software Version 8 (GraphPad Software). No samples were excluded from the analysis. We considered p-values less than 0.05 to be significant, as indicated by *p < 0.05.

## RESULTS

### Reduction in IL-22 production by ILC3 in animals fed an HFD is dependent on VIPergic signaling

**FIGURE 1:**
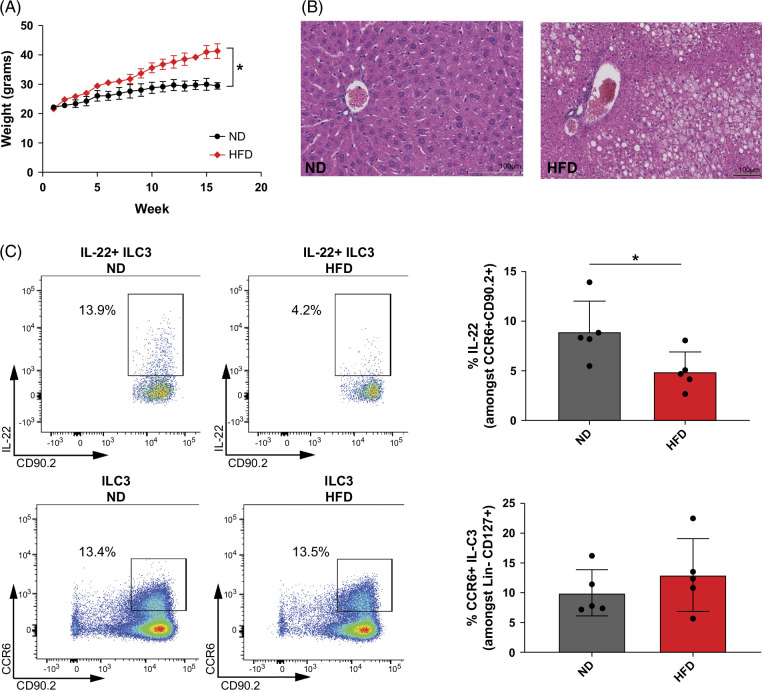
Reduction in IL-22 production by intestinal ILC3 in animals fed an HFD. C57BL/6 mice were fed HFD or ND for 16 weeks before experimentation. (A) Time course of average weight (mg) of mice placed on HFD versus ND for 16 weeks. *Denotes significant weight difference at 16-week time point. Average based on n = 6 in each group. Unpaired nonparametric statistical evaluation with *p < 0.05. Experiment was repeated twice with similar results. (B) Representative image of liver steatosis which is consistently observed at 16 weeks after HFD in our animal facility. (C) (Top row) A significant reduction in the proportion of IL-22^+^ cells among intestinal CCR6+ ILC3 from C57BL/6 mice fed either an HFD or ND for 16 weeks. n = 5 in each group. (Bottom row) No significant differences in % of intestinal CCR6+ ILC3 from C57BL/6 mice fed either an HFD or ND for 16 weeks. n = 5 in each group. Lin– denotes lineage (CD14, CD19, CD11c, CD11b, KLRG1, TCRβ, TCRγ, CD3, and NK1.1)-negative cells. A total of 3 independent experiments showed similar results. Unpaired nonparametric statistical evaluation *p value < 0.05. Abbreviations: HFD, high-fat diet; ILC3, innate lymphoid cells type 3; ND, normal diet.

As a preclinical model of MASLD development, mice were exposed for 16 weeks to an HFD in which fat comprises 60% of the calories. Using this model, we were able to observe significant weight gain and onset of hepatic steatosis at 16 weeks post-HFD initiation (Figures 1A, B). Since the constituents of the diet did not have the addition of fructose, other sugars, or cholesterol, we did not detect features of steatohepatitis on histological examination (ie, lobular inflammation or fibrosis) at 16 weeks.^27^

The protective role of IL-22 in MASLD development, gut permeability, and lipid metabolism has been previously characterized.^5^,^10^ Given that ILC3 is abundant and one of the major cell types producing IL-22 in the intestines,^12^,^28^,^29^ we evaluated whether IL-22 production by these cells is altered in mice under HFD. We observed reduced IL-22 production by intestinal ILC3 isolated from mice exposed for 16 weeks to an HFD compared to a standard chow diet (ND) (Figure 1C and Supplemental Figure S1A, http://links.lww.com/HC9/B37). No differences were observed in the percentages of intestinal CCR6+ ILC3 between ND and HFD mice after 16 weeks of exposure to an HFD (Figure 1C). Importantly, others have shown that ILC3s are not abundant in the liver under homeostatic conditions.^30^,^31^ Congruent with this, we did not observe a change in the frequencies of CCR6+ ILC3 in the livers of mice fed an HFD for 16 weeks (Supplemental Figure S1B, http://links.lww.com/HC9/B37).

**FIGURE 2:**
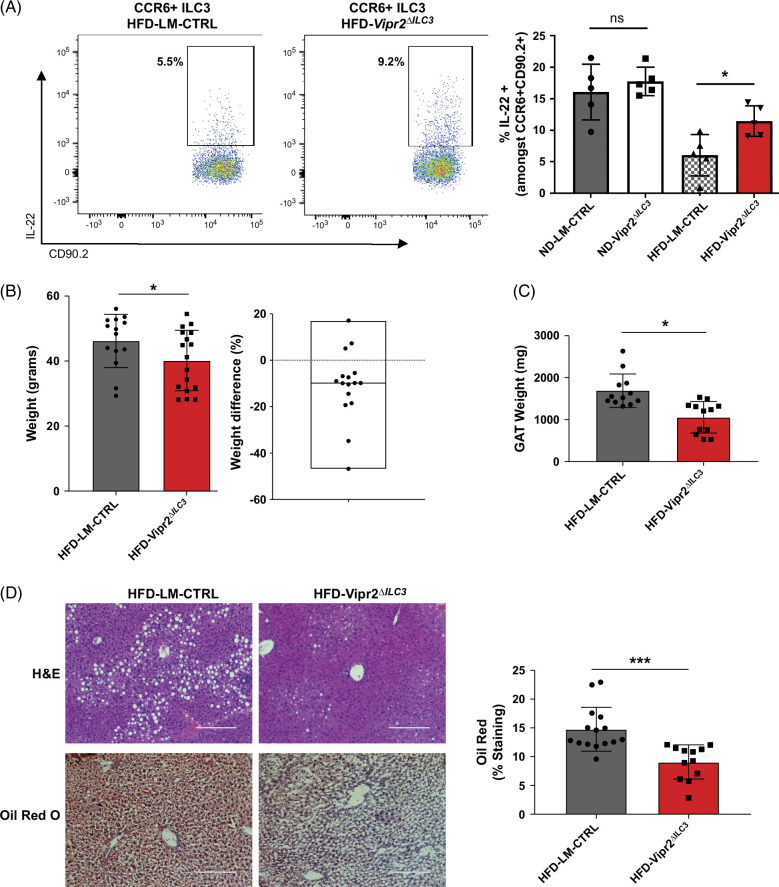
Increased IL-22 production by intestinal ILC3 lacking VIP receptors (*Vipr2* ^*ΔILC3*^) in the setting of HFD is associated with an attenuation of liver steatosis. (A) Littermate control (LM-CTRL) or conditional KO (*Vipr2* ^*ΔILC3*^) mice whereby ILC3 lacked VIP receptors were fed either ND or HFD for 16 weeks. Representative plots showing proportions of intestinal lamina propria IL-22+ ILC3 from *Vipr2* ^*ΔILC3*^ mice compared to LM-CTRL after 16 weeks of HFD or ND. n = 5 in each group. Two independent experiments found similar results. Paired nonparametric statistical evaluation *p value < 0.05. (B) Left: Body weight (g) at 16 weeks of HFD feeding of *Vipr2* ^*ΔILC3*^ and littermate control (LM-CTRL) mice. n = 14 for LM-CTRL, n = 17 for *Vipr2* ^*ΔILC3*^. Of note, the starting weights of *Vipr2* ^*ΔILC3*^ and LM-CTRL mice (~6 weeks of age) were similar with no statistically significant difference. Right: Although the magnitude of total weight gain is higher in this study, the observation of the mean % weight difference shown in the box plot (~10%) in *Vipr2* ^*ΔILC3*^ mice versus LM-CTRL after 16 weeks of HFD is similar to previously reported data of 9-week-old mice fed ND.^18^ Dotted line in box plot demarcates 0% change in weight. Two independent experiments found similar results. *p < 0.05 using paired nonparametric evaluation. (C) GAT weight in male littermate controls (LM-CTRL) and *Vipr2* ^*ΔILC3*^ mice. n = 12 LM-CTRL and n = 12 *Vipr2* ^*ΔILC3*^ mice. Two independent experiments found similar results. *p < 0.05 using paired nonparametric evaluation. All body and tissue weights were collected in a blinded manner. (D) Histological analysis of the liver to evaluate steatosis qualitatively with H&E and quantitatively by lipid staining with Oil Red O. Representative images of histology and Oil Red O quantification with 3 random areas from each individual liver section scored in a blinded fashion (right graph) to account for potential heterogeneity in liver steatosis. n = 5 littermate controls (LM-CTRL), n = 4 *Vipr2* ^*ΔILC3*^. Two independent experiments found similar results. ***p < 0.001 using unpaired nonparametric evaluation. Abbreviations: GAT, gonadal adipose tissue; H&E, hematoxylin and eosin; HFD, high-fat diet; ND, normal diet; VIP, vasoactive intestinal peptide.

We next evaluated whether VIP-mediated inhibition of IL-22 production by intestinal ILC3 contributes to the liver steatosis phenotype in mice fed HFD. We previously showed that IL-22 production by ILC3 is reduced in a VIPR2-dependent manner during feeding (dark period for mice) and that levels of IL-22 increase during fasting (light period for mice).^18^ Indeed, mice with conditional genetic deletion of *Vipr2* in ILC3 (*Vipr2* ^*ΔILC3*^) have normal levels of IL-22 production when compared to their littermates during fasting, and the levels do not reduce during feeding as for wildtype (WT) littermates.^18^ In this study, when samples were collected from mice during the light period, we again did not observe differences in the levels of IL-22 produced by intestinal ILC3 comparing WT littermate controls (LM-CTRL) and *Vipr2* ^*ΔILC3*^ mice on ND. However, we observed higher levels of IL-22 being produced by ILC3 isolated from *Vipr2* ^*ΔILC3*^ mice exposed for 16 weeks to an HFD when compared to LM-CTRL (Figure 2A and Supplemental Figure S1A, http://links.lww.com/HC9/B37). This shows that the reduction of IL-22 production by ILC3 that is induced by exposure to an HFD is dependent, at least in part, on VIP signaling. Of note, despite the expression of RORγt in cells other than ILC3 (eg, T cells), we previously showed that T cells do not express VIPR2 under homeostasis.^18^

### MASLD development is attenuated by the modulation of VIP-producing neurons and ILC3

Since we observed an increase in IL-22 production by intestinal ILC3 in *Vipr2* ^*ΔILC3*^ mice fed an HFD, we then asked whether these mice are protected from the development of hepatic steatosis. After 16 weeks on an HFD, *Vipr2* ^*ΔILC3*^ mice weighed on average ~10% less than littermate controls (Figure 2B). Male *Vipr2* ^*ΔILC3*^ mice also displayed smaller gonadal adipose tissue than their littermate control mice, and both male and female *Vipr2* ^*ΔILC3*^ mice had significant reduction in hepatic steatosis seen on hematoxylin and eosin histology and quantified using Oil Red O staining (Figures 2B–D). We did not identify any difference in glucose tolerance (Intraperitoneal glucose tolerance test), random serum glucose, serum triglycerides, and cholesterol between littermate controls and *Vipr2* ^*ΔILC3*^ mice when samples were collected in fasted mice (Supplemental Figure S1C; top row, http://links.lww.com/HC9/B37), suggesting that this pathway does not prevent alterations in glucose tolerance at 16 weeks after HFD. There were also no differences observed in serum markers of hepatocellular and cholestatic liver injury between WT littermate controls and Vipr2^ΔILC3^ mice (Supplemental Figure S1C; bottom row, http://links.lww.com/HC9/B37).

Both clinical and preclinical studies have implicated the host microbiota in MASLD. Given this, we evaluated whether the attenuated liver steatosis phenotype observed in our Vipr2^ΔILC3^ mice was associated with perturbations in the cecal 16S signatures. Phylogenetic clustering of the microbial communities in the cecal content of WT littermate controls and *Vipr2* ^*ΔILC3*^ mice revealed that the genetic deletion of VIPR2 in ILC3 impacted the microbial composition within the intestine of these mice under ND and HFD (Supplemental Figures S2A–C, http://links.lww.com/HC9/B38). This suggests that differences in the composition of the intestinal microbiota associated with the absence of VIP modulation of ILC3 may be associated with the observed liver phenotype from diet-induced hepatic steatosis.

**FIGURE 3:**
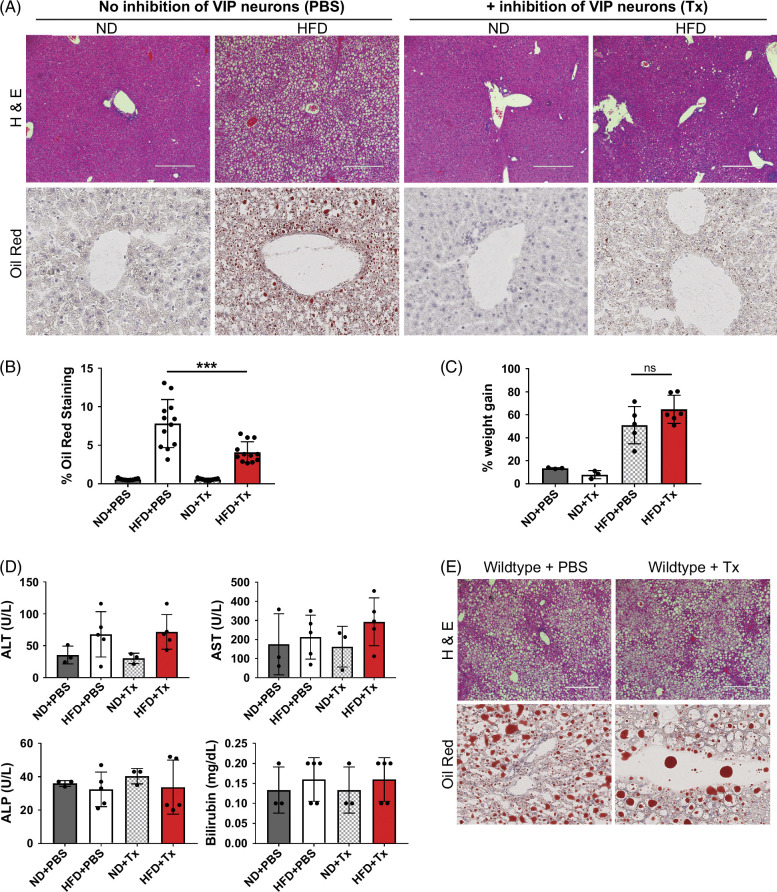
Effect of chemogenetic inhibition of VIP-producing neurons on HFD-induced MASLD. (A) Compound 21 (denoted as Tx; 1 mg/kg) or PBS was injected i.p. under sterile conditions every 48 hours in DREADD mice fed an HFD or ND for 16 weeks. Histology images of liver steatosis were qualitatively evaluated with H&E staining and quantitatively with Oil Red O staining, with 3 random areas from each individual liver section scored in a blinded fashion in (B). ***p < 0.001 using unpaired nonparametric evaluation with n = 4 PBS and n = 4 Compound 21 (Tx)-treated animals. Experiments were repeated twice with similar results. (C) Body weight of DREADD mice at 16 weeks after treatment with Compound 21 (Tx) or control (PBS) while on HFD or ND. ns denotes not statistically significant. (D) Graphs showing serology reflecting hepatocellular liver injury (ALT and AST) and cholestasis (ALP and bilirubin) from DREADD mice with and without Compound 21 (Tx) on either HFD or ND. (E) Histology images of liver steatosis qualitatively evaluated with H&E staining of wildtype (B6 non-DREADD) mice fed an HFD and treated with either Compound 21 (Tx) or control (PBS) for 16 weeks. Statistical analysis in (C) and (D) was done using one-way ANOVA on ranks. Experiments were repeated twice with similar results. Abbreviations: DREADD, Designer Receptors Exclusively Activated by Designer Drugs; H&E, hematoxylin and eosin; HFD, high-fat diet; MASLD, metabolic dysfunction–associated steatotic liver disease; ND, normal diet; VIP, vasoactive intestinal peptide.

Our observation of attenuated liver steatosis with impaired VIP signaling to ILC3 prompted us to evaluate whether the global modulation of VIP-neurons would be feasible in the setting of MASLD. To address this, we expressed a Designer Receptors Exclusively Activated by Designer Drugs (DREADD)-chemogenetic tool (hM4Di) in VIP-producing neurons.^18^,^32^ With this tool, we promoted intermittent inhibition of VIP-producing neurons upon in vivo treatment with the DREADD agonist C21 every other day for 16 weeks during exposure to an HFD. Intermittent inhibition of VIP-producing neurons for 16 weeks resulted in a striking reduction in liver steatosis (Figures 3A, B), suggesting that global intermittent inhibition of VIP signaling could attenuate MASLD. Importantly, this inhibition of VIP-producing neurons did not affect the total body weight as observed in *Vipr2* ^*ΔILC3*^ mice, probably due to the intermittent nature of our protocol (Figure 3C). Moreover, C21-treated and PBS-treated DREADD VIP-Cre mice, on either HFD or ND, were cohoused to minimize the potential confounding batch effects of the commensal microbiota. It is also important to note that the same intermittent chronic treatment with the DREADD agonist C21 did not result in any biochemical evidence of adverse drug induced liver injury or protection against hepatic steatosis (Figures 3D, E) in WT mice. This shows the feasibility of a therapeutic approach centered on intermittent global inhibition of VIPergic activity to attenuate the progression of hepatic steatosis in MASLD.

## DISCUSSION

In this study, we observed that modulation of VIPergic signaling in a neuroimmune interaction can be a potential therapeutic avenue for MASLD. Specifically, both global inhibition of VIP-producing neurons and the targeted removal of the neuroinhibitory control of ILC3 (namely VIPR2) reduced hepatic steatosis in a preclinical model of MASLD induced by an HFD. Both ILC3 and IL-22 have been shown to play critical roles in maintaining intestinal barrier integrity, altering lipid metabolism, and attenuating metabolic imbalance.^10^,^17^,^33^ As such, our findings that *Vipr2* ^*ΔILC3*^ mice under an HFD maintain higher levels of IL-22 production by ILC3 in comparison to WT littermate control mice and concurrently have reduced liver steatosis suggest immune-metabolic interactions are an important therapeutic avenue worth further investigating in MASLD. At 16 weeks under an HFD, we did not appreciate any histological evidence of inflammatory changes, a hallmark of metabolic dysfunction–associated steatohepatitis, in murine livers. This was likely a reflection of both the diet model used and the time frame of diet consumption.^27^,^34^ However, the attenuation of hepatic steatosis is still of great clinical interest, as human genome-wide association studies have linked several gene risk loci associated with the accumulation of excess hepatic lipid content to overall MASLD severity and progression.^35^,^36^,^37^

In our earlier study, we characterized mice with conditional inactivation of *Vipr2* in RORγt^+^ cells and showed that this resulted in altered ILC3-mediated intestinal barrier functions, with reduced lipid absorption in the intestine and enhanced antimicrobial peptide production. There were no changes in cytokine production among RORyt^+^ T lymphocytes, which do not express VIPR2, suggesting that the genetic manipulation in this conditional KO model primarily affects ILC3.^18^ Although IL-22 within the intestinal environment can be produced by various immune cells, including T-helper type 17, T-helper type 22, γδ T cells, and innate lymphoid cells,^12^,^38^,^39^ the selective expression of VIPR2 in ILC3^18^ and our results suggest that specific modulation of ILC3-derived IL-22 could be sufficient to attenuate the development of hepatic steatosis.

Despite previous descriptions of the effects of IL-22 on lipid metabolism, the exact mechanism by which IL-22 could modulate MASLD progression is not fully understood. It is possible that IL-22 contributes to the reduction of hepatic steatosis by impacting metabolism systemically at extraintestinal sites (eg, the liver).^16^,^40^,^41^,^42^ Within the liver environment, we and other groups have observed ILC3 accounts for only 1% of intrahepatic lymphocytes and ~0.2% of total cells.^31^ Present in low numbers within the liver, this may suggest that the attenuation of liver steatosis in *Vipr2* ^*ΔILC3*^ mice could be due to altered ILC3 functions in extrahepatic sites. Interestingly, a recent study demonstrated that IL-22 receptor conditional deletion in intestinal epithelial cells modulates the development of liver steatosis in mice fed HFD. However, an impact on steatosis was not observed when the IL-22 receptor was genetically deleted in hepatocytes.^43^ Taken together, these findings support the hypothesis that the increase of IL-22 production, potentially by intestinal ILC3, and the subsequent tissue response within the intestinal environment might be involved with the attenuation of hepatic steatosis that we observed in our study. This overall supports the important role of the gut-liver axis in steatotic liver disease. Further studies are needed to better understand the extrahepatic tissues and cellular context of VIP, ILC3, and IL-22 signaling as it relates to MASLD. Moreover, intestinal IL-22 impacting intestinal barrier function, altering the host gut microbiota, and regulating the host expression of fat metabolism genes are additional hypotheses that could explain the observed protection in liver steatosis in *Vipr2* ^*ΔILC3*^ mice.^7^,^10^,^18^,^40^,^44^ Indeed, we observed that the composition of the microbiota between WT and *Vipr2* ^*ΔILC3*^ mice are different, as previously described. This raises the possibility that the observed reduction in liver steatosis could be secondary to microbiome perturbations. Additional mechanistic and validation studies are, however, needed to fully characterize the role of these gut microbes in mediating MASLD development.^45^,^46^,^47^,^48^

We observed a marked reduction in hepatic steatosis following intermittent global inhibition of VIP-producing neurons in mice. Although this outcome may be partly driven by increased ILC3-derived IL-22 production in the intestinal mucosa, we cannot rule out IL-22 and ILC3-independent effects in these animals. Within the liver environment, VIP itself has been described to attenuate ischemic liver injury.^49^ Importantly, our finding brings to light an intriguing hypothesis that alterations in peripheral neuronal functions might contribute to immune and metabolic dysfunctions associated with the development of metabolic syndrome.

In our previous publication, we did not observe a reduction in IL-22 production in ILC3 from *Vipr2* ^*ΔILC3*^ mice during the fasting period when compared to WT littermates. Differences only arise during periods that animals are actively eating, which is when VIP-producing neurons are active and releasing VIP in the intestine.^18^ Here, we reproduce these observations of no difference in IL-22 production during the resting/fasting period (light period) for *Vipr2* ^*ΔILC3*^ mice and littermate WT mice under an ND. However, for mice under an HFD, when collected during the resting/fasting period (light period), we observed a marked reduction in IL-22 production in ILC3 isolated from littermate WT mice versus *Vipr2* ^*ΔILC3*^ mice. Since activation of VIP-producing neurons in the intestine has been shown to be triggered by food consumption, our results could reflect previous observations of an altered diurnal pattern of feeding behavior in mice under an HFD, namely, an increase in food consumption during the light period, which is not present in mice under an ND.^50^ Currently, the dietary signals that trigger VIP release in the intestine are unknown. Whether increased fat content in the diet or alterations in the diurnal pattern of feeding directly promotes increased and continuous VIP release in the intestine is unclear. Further characterization of how dietary perturbations affect the activity of VIP-producing neurons might reveal new therapeutic approaches for the modulation of metabolic disorders. Furthermore, ILC3 activation and IL-22 expression are reduced with the aging of mice.^17^ Indeed, WT and *Vipr2* ^*ΔILC3*^ mice analyzed in the present study, which were around 22–24 weeks old, displayed lower levels of IL-22 production when compared to our previous observations in mice 6 to 12 weeks old.^18^ Nevertheless, we see a further reduction of IL-22 production in our older WT mice under an HFD when compared to older mice on an ND. This suggests that diet and age-related immune changes could synergize in an environment that further reduces ILC3 functions and the progression of MASLD. As such, dedicated future endeavors evaluating the complex relationship between aging and physiological changes in the host and ILC3 function are needed.

In conclusion, our findings highlight the potential for targeting VIP signaling and ILC3 as a novel therapeutic strategy to ameliorate hepatic steatosis and highlight the need to further understand how neuroimmune modulation can affect metabolic disorders, including MASLD. These results also underline the importance of evaluating early immune-metabolic events within the intestinal microenvironment to gain a better understanding of how gut perturbations can subsequently mediate the development of liver disease.

## Supplementary Material

**Figure:**
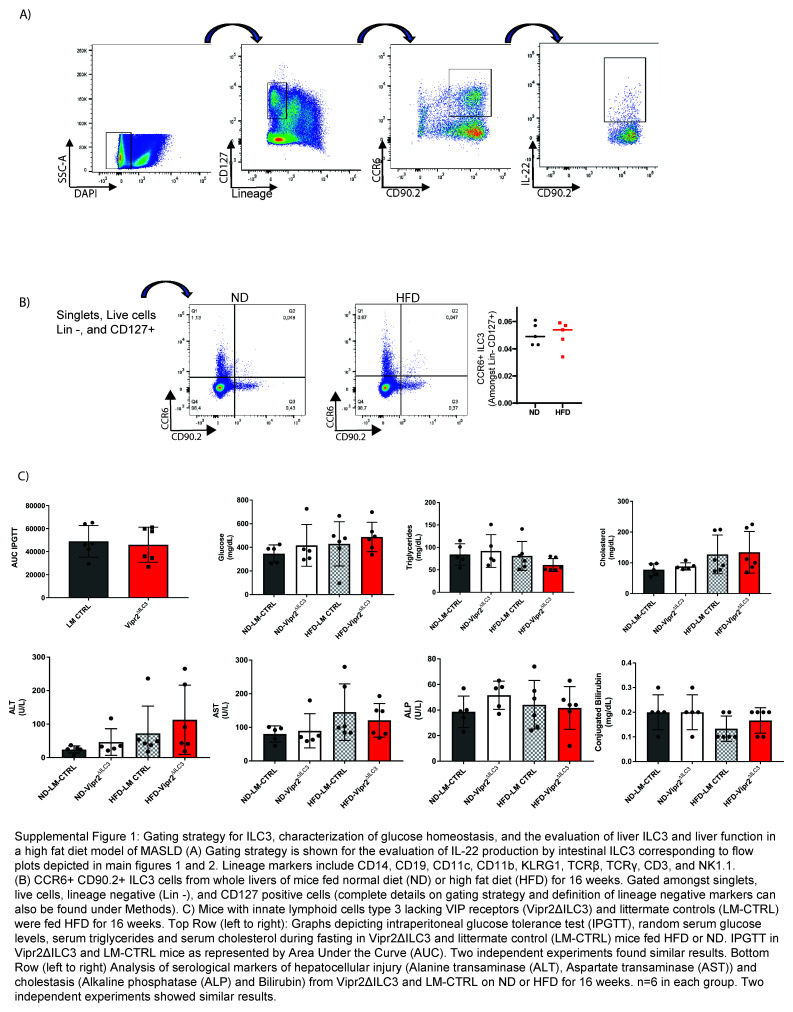


**Figure:**
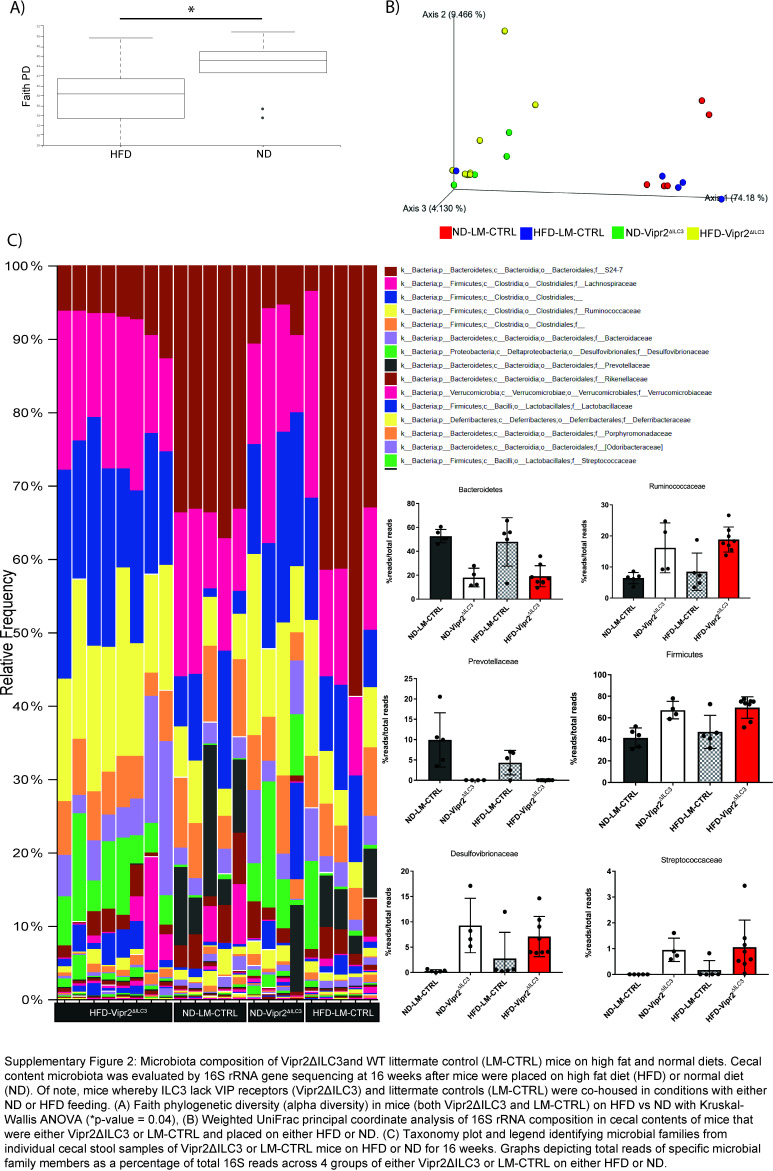


## Data Availability

Data and analytic methods will be made available to other researchers upon request. Henry H. Nguyen, Jhimmy Talbot, and Dan R. Littman designed the study and analyzed the data. Henry H. Nguyen performed the experiments with the assistance of Jhimmy Talbot, Dayi Li, and Varsha Raghavan. Henry H. Nguyen and Dan R. Littman wrote the manuscript with edits contributed by Jhimmy Talbot. Dan R. Littman supervised the research.
